# Multiple myeloma with simultaneous cutaneous and central nervous system involvement

**DOI:** 10.1002/ccr3.1626

**Published:** 2018-09-05

**Authors:** James Nguyen, Hani Hassoun

**Affiliations:** ^1^ Memorial Sloan‐Kettering Cancer Center New York NY USA

**Keywords:** leptomeningeal disease, multiple myeloma, skin malignancy

## Abstract

Cutaneous involvement in multiple myeloma is an extremely rare occurrence that is underrecognized and akin CNS involvement, typically occurs late in the disease course, and is associated with an aggressive biology. Pathologic examination is key to the diagnosis and a standard of care treatment has not been established for these patients.

A 60‐y‐old gentleman presented with complaints of fever and right shoulder pain. A complete evaluation showed extensive lytic lesions involving the right clavicle, sternum, cervical and thoracic spine, as well as adjacent multiple soft tissue masses. A biopsy of the right clavicular mass revealed an anaplastic plasmacytoma with kappa light chain restriction and a MIB1 index of 60%. Serum protein electrophoresis demonstrated a monoclonal IgG kappa, with an IgG level of 2.1 g/dL. The patient received radiation therapy (5000 cGy) to the right shoulder with improvement in symptoms and proceeded with induction therapy consisting of 4 cycles of lenalidomide and dexamethasone with achievement of a very good Partial Response. However, shortly after completion of this treatment, the patient complained of recurrent fevers and a bone marrow biopsy revealed sheets of plasma cells. Planned ASCT was postponed and further chemotherapy consisting of 3 cycles of CVAD (Cytoxan, vincristine, Adriamycin, and dexamethasone) was administered. At the completion of this treatment, a bone marrow biopsy demonstrated <3% of PCs and the patient was scheduled for stem cell mobilization with Cyclophosphamide and G‐CSF. On the day of admission, he was found to have new right upper extremity weakness and a papular rash (Figure 1A) on the chest. Evaluation of the weakness including MRI and CT scan of the head showed no significant findings. Cerebrospinal fluid examination demonstrated the presence of multiple plasma cells (Figure 1B) with Kappa light chain restriction. Biopsy of a chest papule showed infiltration of the skin by plasma cells (Figure 1C) with kappa light chain restriction as well (Figure 1D). The patient declined further treatment and expired shortly after from disease progression.

**Figure 1:**
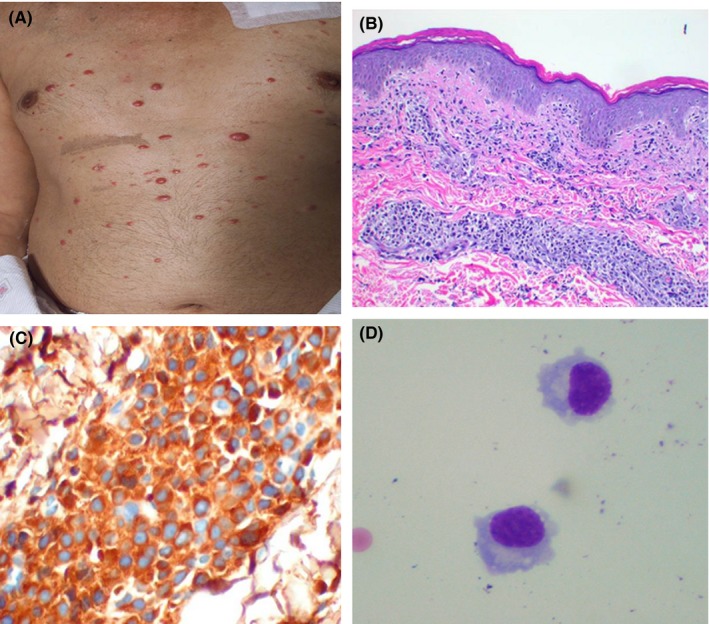
On the day of admission, the patient was found to have new right upper extremity weakness and a papular rash (A) on the chest. Biopsy of a chest papule showed infiltration of the skin by plasma cells (B) with kappa light chain restriction (C). Evaluation of the weakness including MRI and CT scan of the head showed no significant findings. Cerebrospinal fluid examination demonstrated the presence of multiple plasma cells with Kappa light chain restriction (D)

Cutaneous involvement is a rare manifestation of multiple myeloma (MM). It usually occurs late in the course of the disease; however, a recent study suggests that when cutaneous involvement is present earlier in the course of the disease, it reflects a more aggressive form of MM.^1^ The most common sites involved by order of decreasing frequency are the trunk, scalp, face, neck, lower extremities, and upper extremities. A standard of care has not been established for myeloma patients with cutaneous involvement. Many treatments, including novel agents and stem cell transplantation, have shown only transient activity. Patients succumb to their disease, on average, within 8.5 mo of cutaneous involvement. Most of them have extensive plasmacytic infiltration of multiple organs at autopsy, thus supporting the growing notion in the field that cutaneous involvement reflects an aggressive biology.

CSF involvement in MM is also rare and believed to occur in roughly 1% of all cases.^2^ Similar to cutaneous involvement, CSF involvement occurs most frequently in the setting of advanced disease stages and is associated with a poor prognosis. A multi‐modality treatment approach using combined cranial/spinal irradiation, intrathecal chemotherapy, in addition to an oral immunomodulatory agent may have promising clinical activity for CSF involvement in MM.^3^ However, treatment options for CSF involvement remain limited and have also shown to have only transient efficacy, attesting to the aggressive biology of the disease.

Interestingly, CSF involvement in association with cutaneous involvement, as seen in this patient, has been reported in very few patients with MM. The unusual association of cutaneous and CNS involvement in MM would portend an extremely poor prognosis. Despite the aggressive behavior demonstrated by the reported cases of simultaneous cutaneous and CSF involvement in MM, a standard of care treatment has yet to be established.

## CONFLICT OF INTEREST

None declared.

## AUTHORSHIP

JN: conceived and designed the report, analyzed the data, wrote and reviewed the manuscript and approved the final draft. HH: conceived and designed the report, collected the data, analyzed the data, wrote and reviewed the manuscript and approved the final draft.
